# Outcomes of radiotherapy in advanced external auditory canal cancer

**DOI:** 10.1093/jrr/rrz010

**Published:** 2019-04-11

**Authors:** Takuya Nagano, Ryo-ichi Yoshimura, Mio Kojima, Keiko Nakagawa, Kazuma Toda

**Affiliations:** Department of Radiation Therapeutics and Oncology, Tokyo Medical and Dental University, 1-5-45 Yushima, Bunkyo-ku, Tokyo, Japan

**Keywords:** external auditory canal cancer, radiotherapy, chemotherapy, radical surgery

## Abstract

External auditory canal cancer (EACC) is a rare malignant tumor. In the present study, we retrospectively evaluated the treatment results in patients with advanced EACC who were treated using external-beam radiotherapy (EBRT) combined with chemotherapy or radical surgery. Overall, 21 patients with Stage III (*n* = 8) or Stage IV (*n* = 13) EACC who underwent initial treatment at our hospital between 2003 and 2016 were enrolled in this study. The 2-year overall survival (OS) and locoregional control (LRC) rates of all patients were 62% and 71%, respectively. The 2-year OS and LRC rates in patients who had received EBRT and concurrent chemotherapy with docetaxel, cisplatin and 5-fluorouracil (TPF, *n* = 6) were 100%. These results were higher than the 2-year OS and LRC rates of 62% and 69%, respectively, in patients who had received radical surgery and EBRT (*n* = 13). The rates were 0% in those who had neither received TPF nor undergone surgery in addition to EBRT (*n* = 2). Grade 3 bone or soft tissue necrosis was observed in 2 patients who had undergone surgery and postoperative EBRT. Our data suggest that the combination therapy of EBRT and surgery and/or chemotherapy may be the most effective treatment options for advanced EACC, and EBRT with concurrent chemotherapy with TPF is potentially the most acceptable.

## INTRODUCTION

External auditory canal cancer (EACC) is a rare malignant tumor that affects 1–6 per 1 million population-years [1, 2]. The low numbers of patients in studies to date have led to an appropriate treatment strategy for EACC remaining controversial. Several clinical reviews and meta-analyses have concluded that radical primary surgical treatment combined with postoperative radiotherapy should be recommended as the standard of care for advanced EACC [3–5]. However, radical surgery, including subtotal temporal bone resection and total temporal bone resection, can cause severe complications, such as meningitis, cerebrospinal fluid leak, encephalitis, brain infarction, deafness, and facial palsy [6].

Although certain study groups have reported the results of chemoradiotherapy as a non-surgical treatment, and Shinoyama *et al.* [7] have reported a better prognosis with chemoradiotherapy than with other modalities, such as surgery with/without external-beam radiotherapy (EBRT), EBRT alone, or proton therapy, the number of studies are still limited.

At our institute we have treated patients with EACC with EBRT and radical surgery and/or concurrent chemotherapy. In the present study, we aimed to retrospectively evaluate the treatments and compare their outcomes.

## MATERIALS AND METHODS

### Patients

We reviewed the irradiation records and extracted the data of patients who received initial definitive treatment including EBRT for EACC between 2003 and 2016. The medical records were reviewed to calculate survival duration and locoregional control (LRC) and to evaluate toxicity after EBRT. In the present study, the EACCs of all enrolled patients were pathologically diagnosed as squamous cell carcinoma, and they were restaged according to the Pittsburgh staging system [8], based on clinical examination, computed tomography (CT), magnetic resonance imaging (MRI), and/or positron emission tomography (PET) at the initial diagnosis.

This study was approved by our institutional review board in February 2017 (Approval No. M2016–272).

### Therapy

All patients enrolled in the present study received EBRT, which was planned by the radiation therapy planning system based on CT images, and 3D conformal radiation therapy (3D-CRT) or intensity-modulated radiation therapy (IMRT) was performed. Definitive EBRT without surgery or EBRT before planned surgery (preoperative EBRT) and/or after surgery (postoperative EBRT) were performed.

In cases of preoperative EBRT or definitive EBRT, the primary tumor visualized on CT, MRI and/or PET-CT images was delineated as the gross tumor volume (GTV); the external auditory canal (EAC) including the GTV and surrounding tissues excluding risk organs (brain, brain stem, etc.) were delineated as the clinical target volume (CTV), and the CTV plus a margin of 5 mm was delineated as the planning target volume (PTV). For patients who received postoperative EBRT, the tumor bed as visualized on CT, MRI and/or PET-CT images obtained before surgery and the region of tumor infiltration diagnosed pathologically were delineated as the CTV, and the CTV plus a margin of 5 mm was delineated as the PTV. The lymph node area was not included in the target volume of patients without lymph node metastasis or of patients receiving preoperative EBRT. In cases of definitive EBRT or postoperative EBRT, and patients with lymph node metastasis, the CTV included the lymph node level of metastasis and neighboring lymph nodes. The CTV of the lymph node area plus 5 mm was included in the PTV.

Whether chemotherapy was administered at all and what chemotherapeutic regimen was employed were determined according to the patient’s general condition and the treatment policy at that time: while chemotherapy was not routinely used for EACC before 2014, chemotherapy with the combination regimen of docetaxel, cisplatin and 5-fluorouracil (TPF) was introduced for concurrent administration with definitive EBRT for head-and-neck cancer, including EACC, at our hospital in 2014.

As a surgery, subtotal temporal resection or lateral temporal bone resection with reconstruction was performed, and neck dissection was performed for patients who had cervical lymph node metastasis.

### Toxicity

Toxicity can occur as a result of surgery, chemotherapy and EBRT. The occurrence of facial paralysis is usually as a sequela of surgery, and myelosuppression and electrolyte imbalance is usually caused by chemotherapy. In the present study, we extracted the late toxicities associated with EBRT, and classified them according to the Common Terminology Criteria for Adverse Events version 4.0 [9].

### Follow-up and analysis

Following the completion of EBRT, the patients were examined by the head-and-neck surgeon, plastic surgeon, and/or radiation oncologist. The follow-up interval varied among the patients depending upon the condition of each. Recurrence in the head-and-neck region, including the EAC, surrounding tissues, and lymph nodes, was defined as locoregional recurrence. Recurrence was diagnosed by imaging techniques, such as CT, MRI and/or PET-CT, and divided into in-field recurrence that occurred in the PTV and out-of-field recurrence that occurred outside the PTV.

The duration of overall survival (OS) and LRC from the start of treatment was calculated by the Kaplan–Meier method and evaluated according to the clinical factors and treatment factors using the log-rank test. Differences were considered statistically significant for *P* < 0.05. Statistical analysis was performed with IBM SPSS Statistics.

## RESULTS

### Patient data

Overall, 23 patients with EACC received EBRT during the specific period; however, 2 patients were excluded because they had been treated with Cyberknife to prevent the recurrence of EACC; the remaining 21 patients were enrolled in the present study. There were 13 women and 8 men, and their age ranged from 39 to 71 years, with a median of 57 years. Among the patients, 95% (20/21) had T3 or T4 EACC, and 29% (6/21) had cervical lymph node metastasis. Eight patients were in Stage III, and the other 13 patients were in Stage IV (Table 1).

**Table 1.** Tumor and treatment factors

Factors	
Total	21
Age	
Range	39–71 years
Median	57 years
Sex	
Female	13
Male	8
T classification	
1	0
2	1
3	10
4	10
N classification	
0	15
1	5
2	1
3	0
Stage	
III	8
IV	13
EBRT dose	
Range	30–70 Gy
Median	50 Gy
EBRT technique	
3D-CRT (orthogonal irradiation)	19
IMRT	2
Chemotherapy	
No	9
TPF	6
TS-1	3
CDDP	1
DTX	1
UFT	1
Surgery	
Subtotal temporal resection	6
Lateral temporal bone resection	4
Unknown	3
No	8
Surgical margin	
Positive	3
Negative	10

TPF = docetaxel + cisplatin + 5-fluorouracil; TS-1 = tegafur/gimeracil/oteracil; CDDP = cisplatin; DTX = docetaxel; UFT = tegafur/uracil; 3D-CRT = 3D conformal radiation therapy; IMRT = intensity-modulated radiation therapy; EBRT = external-beam radiation therapy.

The treatment methods are summarized in Table 2. Surgery (namely, lateral or subtotal temporal bone resection) was performed in 13 patients.

**Table 2.** Therapy

Therapy	Stage (*n*)	
	III	IV
EBRT alone	0	1
EBRT w/TPF	2	4
EBRT w/DTX	0	1
preEBRT—surgery	1	0
preEBRT w/UFT—surgery	1	0
preEBRT w/TS-1—surgery	0	1
preEBRT—surgery—postEBRT	1	1
preEBRT—surgery—postEBRT w/TS-1	0	1
Surgery—postEBRT	1	4
Surgery—postEBRT w/cisplatin	1	0
Surgery—postEBRT w/TS-1	1	0

w/ = with; — = then; UFT = tegafur/uracil; TS-1 = tegafur/gimeracil/oteracil; EBRT = external-beam radiation therapy; preEBRT = preoperative EBRT; postEBRT = postoperative EBRT; TPF = docetaxel + cisplatin + 5-fluorouracil; DTX = docetaxel.

Chemotherapy was administered in 57% of all patients (12/21). Approximately half of these patients (6/12) received TPF, 3 patients had tegafur–gimeracil–oteracil potassium (TS-1), 1 had cisplatin, 1 had docetaxel, and 1 had tegafur–uracil (UFT).

EBRT using 3D-CRT was employed in 19 patients, including using the orthogonal beam technique in 14 patients, rotation beam technique in 3 patients, three-field technique in 1 patient, and one-field technique in 1 patient; the remaining 2 patients received IMRT. The energy of the irradiating X-ray beam was 4 MV in 18 patients and 6 MV in 3 patients. The radiation dose for preoperative EBRT was 30 Gy (*n* = 3) or 40 Gy (*n* = 3), and that for the postoperative EBRT dose was 20 Gy (*n* = 2) or 30 Gy (*n* = 1) in patients who had received preoperative EBRT and 50 Gy (*n* = 6) or 60 Gy (*n* = 1) in patients who had not received preoperative EBRT. Postoperative EBRT was administered in 10 patients on the basis of the pathological findings: parotid gland infiltration in 6 patients, mandibular bone infiltration in 2 patients, and positive surgical margin in 2 patients. The definitive radiation dose for EBRT in the remaining 8 patients who did not undergo surgery ranged from 40 to 70 Gy (median 60 Gy), including 40 Gy in 1 patient who discontinued EBRT because of tumor progression detected even during the EBRT, 60 Gy in 5 patients, and 70 Gy in the remaining 2 patients.

The follow-up duration of all patients ranged from 7 to 158 months (median 23 months), whereas the duration excluding deceased patients ranged from 11 to 158 months (median 52 months).

### Outcomes

**Figure 1.**
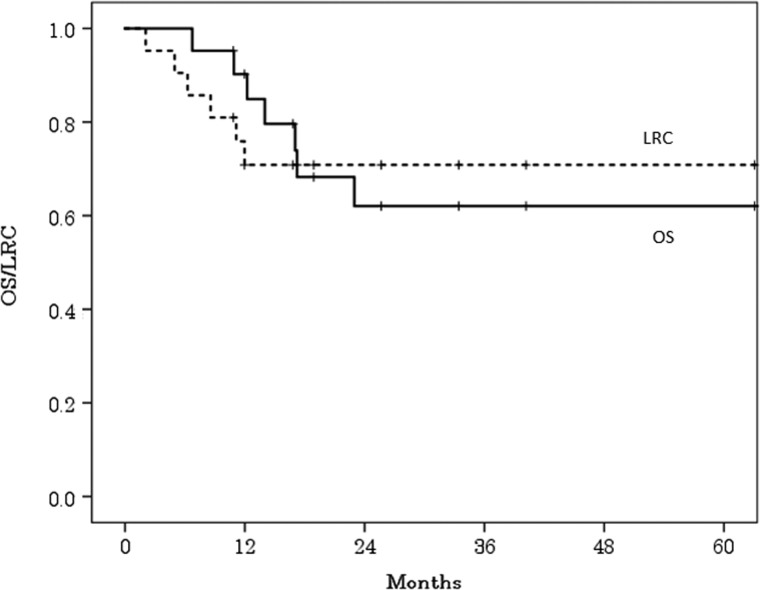
Overall survival (OS) curve and locoregional control (LRC) curve for all patients.

The 2-year OS and LRC rates of all patients were 62% [95% confidence interval (CI), 40–85%] and 71% (95% CI, 51–91%), respectively (Fig. 1). No clinical factors (age, sex, T-stage, N-stage, or clinical stage) were significantly associated with OS or LRC rates, although OS and LRC rates of younger patients were lower than those of the older patients, and LRC rate decreased with increasing cT-stage (Table 3).

**Table 3.** Treatment outcome according to the clinical and treatment factors

Factors	*n*	Overall survival		Locoregional control	
		2-year rate	*P*	2-year rate	*P*
		[95% CI (%)]		[95% CI (%)]	
Age (years)					
≤60	16	48 (21–75)		61 (37–86)	
>60	5	100	0.07	100	0.1
Sex					
Female	13	56 (27–85)		68 (41–94)	
Male	8	73 (41–100)	0.7	75 (45–100)	0.8
T classification					
2	1	100		100	
3	10	75 (45–105)	0.3	90 (71–100)	0.2
4	10	44 (10–78)		50 (19–81)	
N classification					
0	15	57 (31–83)		66 (41–90)	
1	5	80 (45–100)	0.8	80 (45–100)	0.8
2	1	100		100	
Clinical stage					
III	8	71 (38–100)		88 (65–100)	
IV	13	56 (25–86)	0.5	62 (35–88)	0.3
Surgery					
No	8	73 (41–100)		75 (45–100)	
Yes	13	62 (35–88)	0.9	69 (44–94)	0.9
Chemotherapy					
No	9	44 (12–77)		56 (23–88)	
Non-TPF	6	67 (29–100)	0.3	67 (29–100)	0.2
TPF	6	100		100	
EBRT					
≤50 Gy	11	55 (25–84)		64 (35–92)	
>50 Gy	10	76 (47–100)	0.5	80 (55–100)	0.5

EBRT = external-beam radiotherapy; TPF = docetaxel, cisplatin and 5-fluorouracil; CI = confidence interval.

According to the treatment modality, no significant difference was found in OS or LRC rates between patients who received surgery (*n* = 13) and patients who did not receive surgery (*n* = 8), between patients who received chemotherapy (*n* = 12) and patients who did not receive chemotherapy (*n* = 9), or between patients who received an EBRT dose of >50 Gy (*n* = 11) and patients who received EBRT dose of ≤50 Gy (*n* = 10). Regarding the influence of the chemotherapy regimen used, the OS and LRC rates of patients who received TPF (*n* = 6) were longer than those who received chemotherapy with other chemotherapeutic regimens (*n* = 6) or no chemotherapy (*n* = 9), although the differences were not significant (Table 3).

According to the treatment combination, trimodality treatment of surgery, chemotherapy, and EBRT was performed in 5 patients; the combination of surgery and EBRT was performed in 8 patients; the combination of chemotherapy and EBRT was performed in 7 patients; and EBRT alone was performed in 1 patient. The 2-year OS and LRC rates of patients who received trimodality treatment were 80% (95% CI, 45–100%) and 80% (95% CI, 45–100%), respectively, those of patients who received a combination of surgery and EBRT were 50% (95% CI, 15–85%) and 63% (95% CI, 29–96%), respectively, and those of patients who received a combination of chemotherapy and EBRT were 83% (95% CI, 54–100%) and 86% (95% CI, 60–100%), respectively. Patients who received EBRT alone showed local disease progression at 2 months after the start of the treatment.

**Figure 2.**
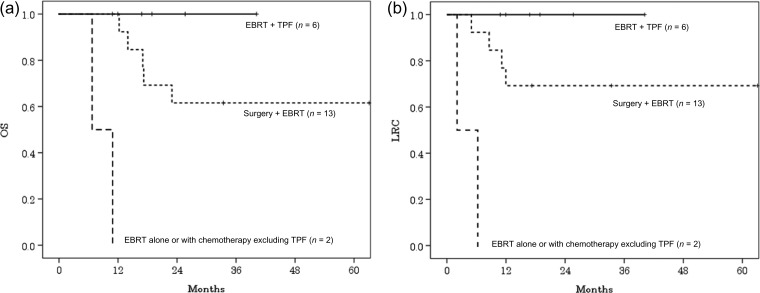
(a) Overall survival (OS) curves of patients with external auditory canal cancer (EACC) treated using external-beam radiotherapy (EBRT) and docetaxel, cisplatin and 5-fluorouracil (TPF); surgery and EBRT; and EBRT alone or with chemotherapy excluding TPF. (b) Locoregional control (LRC) curves for patients with external auditory canal cancer (EACC) treated using external-beam radiotherapy (EBRT) and docetaxel, cisplatin and 5-fluorouracil (TPF); surgery and EBRT; and EBRT alone or with chemotherapy excluding TPF.

In the patients who received a combination of chemotherapy and EBRT, the 2-year OS and LRC rates of patients who received TPF and EBRT (*n* = 6) were 100% and 100%, respectively. These results were better than those of patients who received surgery and EBRT with or without chemotherapy [*n* = 13; the 2-year OS and LRC rates were 62% (95% CI, 35–88%) and 69% (95% CI, 44–94%), respectively], and also better than those of patients who did not receive TPF or surgery (*n* = 2; the 2-year OS and LRC rates were 0% and 0%, respectively). There was a significant difference in the OS and LRC between the three groups [OS, *P* < 0.001 (Fig. 2a); LRC, *P* < 0.001 (Fig. 2b)]; however, the differences in the OS and LRC between the patients who underwent surgery and EBRT and those who underwent TPF and EBRT was not significant (OS, *P* = 0.2; LRC, *P* = 0.2). The EBRT dose differed significantly between the above two groups, and there was a borderline difference in the follow-up duration (Table 4).

**Table 4.** Characteristics distinguishing patients who had received EBRT combined with surgery from patients who had received EBRT and chemotherapy with TPF

		EBRT combined with		
		Surgery (*n*)	TPF (*n*)	*P*
Total		13	6	
Age (years)	≤60/>60	9/4	5/1	0.5
Sex	F/M	9/4	3/3	0.4
T classification	2/3/4	1/7/5	0/3/3	0.7
N classification	0/1–2	11/2	3/3	0.1
Clinical stage	III/IV	6/7	2/4	0.6
EBRT dose	≤50 Gy/>50 Gy	10/3	0/6	0.02
Follow-up	Range (months)	11–157	11–40	0.07
	Median (months)	61	18	

### Recurrences

Local recurrence was found in six patients, two recurrences occurred in-field and the other four recurrences occurred out-of-field: two of the latter recurrences in the parotid, one at the skull base, and one in the masticator space. One in-field recurrence and three out-of-field recurrences were found in patients who had received surgery, and one in-field and one out-of-field recurrences were seen in patients who did not receive surgery.

The two patients with in-field recurrences received chemotherapy, followed by best supportive care, and the patients with out-of-field recurrences received chemotherapy (*n* = 2), stereotactic radiotherapy (*n* = 1), or stereotactic radiotherapy and additional surgery (*n* = 1). However, all patients died between 4.7 and 16 (median, 5.4) months after the find of recurrences.

### Radiation-induced toxicity

All patients who underwent surgery suffered from facial nerve disorder, and three of these patients underwent repeated cosmetic surgery, including eyebrow lift. Moreover, 57% of patients who received chemotherapy suffered from neutropenia and leukocytopenia of Grade 3.

Eight radiation-induced late toxicities were seen in seven patients. Bone or soft tissue necrosis around the ear was noted in three patients, osteonecrosis of the jaw in three, and stenosis of the EAC in two (Table 5).

**Table 5.** Relationship between treatment and radiation-induced toxicity

Toxicity	Grade	EBRT alone	EBRT + Ch	S + EBRT	S + EBRT + Ch
Osteonecrosis of the jaw	1	0	2	0	1
EAC stenosis	1	0	2	0	0
Brain necrosis	1	0	1	0	0
Bone or soft tissue necrosis	2	0	1	0	0
	3	0	0	2	0

EAC = external auditory canal; EBRT = external-beam radiotherapy; Ch = chemotherapy; S = surgery.

All three patients who suffered from bone or soft tissue necrosis had Stage IV EACC. One of them had received combined treatment with 60 Gy of EBRT and TPF, while the other two patients underwent surgery and 50 Gy of postoperative EBRT. One of two patients with Grade 3 soft tissue and bone necrosis had undergone CyberKnife radiotherapy for recurrence following surgery and postoperative EBRT. The remaining patient suffered from Grade 3 soft tissue necrosis after surgery and postoperative EBRT, and underwent skin grafting.

## DISCUSSION

In the present study, the 2-year OS rate of all EACC patients who received EBRT was 62% and that of Stage III and IV patients was 71% and 56%, respectively; these rates were similar to those reported in prior studies or a little better than them. A meta-analysis showed that the 5-year OS rates of Stage III and IV EACC patients were 75.4% and 35.8%, respectively, and that the 5-year OS rate of patients who did not undergo surgery (EBRT alone, chemotherapy alone, or chemoradiotherapy) was only 29% [6]. A multi-institutional retrospective review in Japan showed that the 5-year disease-free survival rate for T3 EACC patients was 25%, and the rates of patients who received surgery and EBRT and EBRT alone were 46% and 0%, respectively [4]. Comparing our 2-year OS rate with Ogawa *et al.*’s 5-year OS rate may be difficult, but long-term outcomes by Mazzoni *et al.* [10] and Leong *et al.* [11] showed there was no remarkable change in the survival curve for EACC patients after 2 years from treatment.

There was an impressive result in our study: the 2-year OS rate of the eight patients who did not receive surgery was 73% (which was higher than the rate found in past studies) [4, 6]. Among patients who did not receive surgery, the prognosis for patients who received a combination therapy of EBRT and concurrent chemotherapy with TPF was better than that of other patients. Only a few studies have reported treatment results of combination therapy of EBRT and concurrent chemotherapy with TPF for EACC patients. Shiga *et al.* [12] reported the results for nine Stage IV EACC patients who received EBRT and TPF, and the 2-year OS rate was 57%. Shinoyama *et al.* [7] treated 10 Stages III and IV EACC patients who received EBRT and TPF and reported that the 2-year OS rate was 70%. In the current study, the 2-year OS rate, and also the 2-year LRC rate, for the six patients who received EBRT and TPF was 100%, higher than the rates for 13 patients who received surgery and EBRT with/without chemotherapy involving chemotherapeutic drugs other than TPF. However, the difference was not statistically significant, and it might have been attributable to the significant difference in the EBRT dose and the borderline significant difference in the observation period between the patients who received EBRT and TPF (for whom the medians of the EBRT dose and the observation period were 60 Gy and 18 months, respectively) and those who received EBRT and surgery (for whom the medians were 50 Gy and 61 months, respectively).

Katori *et al.* [13] have compared induction chemotherapy with TPF followed by EBRT and concurrent chemoradiotherapy with TPF in patients with advanced squamous cell carcinoma of the head and neck and found that with respect to OS, concurrent chemoradiotherapy with TPF was better than induction chemotherapy with TPF followed by EBRT, but in the group who received concurrent chemotherapy with TPF, Grade 3 or 4 mucositis was observed in 79% (40% in the group that received induction TPF followed by EBRT), and Grade 3 or 4 leukocytopenia was observed in 53% (40% in the group who received induction TPF followed by EBRT), although their study included no EACC patients. In our study of EACC, 4 out of 6 (67%) patients who received EBRT and TPF suffered from five late toxicities induced by irradiation. One patient, with Stage III EACC, received concurrent EBRT at 70 Gy and TPF and suffered from Grade 1 EAC stenosis. The second patient, with Stage IV EACC, received EBRT at 60 Gy and TPF and suffered from Grade 2 soft tissue necrosis and Grade 1 EAC stenosis. The remaining two patients with Stage IV EACC who received EBRT at 60 Gy and TPF developed Grade 1 osteoradionecrosis. Although administration of TPF chemotherapy requires experience in delivery of the drugs and provision of sufficient supportive care to avoid hematological toxicity [13], the severe mucositis that often occurs in the irradiation field in patients with other head-and-neck cancers may not occur in patients with EACC, and radiation-induced toxicity in patients with EACC is considered to be within acceptable limits.

Although brain injury has been seen with radical radiotherapy doses of 66 Gy in 2 Gy per fraction, Pemberton *et al.* [14] have reported that doses of ≥66 Gy for primary radical radiotherapy for middle ear cancer and EACC are deemed necessary because lower doses are associated with increased recurrence. In the present study, EBRT at ≤50 Gy was administered for patients following radical surgery. Our findings suggest that a dose of 60 Gy or more may be necessary, despite surgery, to improve the OS and LRC rates, but attention needs to be paid to the EBRT field. We observed out-of-field recurrence in 23% (3/13) of patients who received EBRT following radical surgery, of which 2 patients had negative surgical margins, even though Yin *et al.* [15] have reported in a multi-institutional study that a free resection margin improves the survival rate. It is difficult to delineate the target after surgery in spite of the available data of images of tumor spread before surgery, the surgical record, and the pathological report. A wide field that would include surrounding tissues while avoiding the organs at risk may be necessary with use of the IMRT technique, which has obvious benefits in target volume conformation.

Because this present study was retrospective and the treatment policy has changed with time, it appears that there could have been a bias in patient selection. Moreover, the observation period of patients who received EBRT and TPF was shorter than that of the patients who underwent EBRT and surgery, and further long-term follow-up may be necessary for a reasonable comparison between the two treatment strategies.

In conclusion, our data suggest that the combination therapy of EBRT and surgery and/or chemotherapy may be the most effective treatment options for advanced EACC, and EBRT with concurrent chemotherapy with TPF is potentially the most acceptable. However, further investigation is needed to determine the optimal dose and the appropriate field for EBRT in patients with EACC. Furthermore, because of the limited number of patients with this cancer, a multicenter study would be desirable.
